# Oral Lesions With Identical Clinical Presentation and Different Histopathological Diagnoses: A Case Series of Mucocele, Schwannoma, and Hamartoma

**DOI:** 10.7759/cureus.93203

**Published:** 2025-09-25

**Authors:** Cristina Suaza, Lenin Torres-Osorio, Jaime E Plazas Román, Adel Martinez Martinez, Antonio Diaz, Carlos M Ardila

**Affiliations:** 1 Private Practice, Dentica Clinic, Bogotá, COL; 2 School of Dentistry, University of Cartagena, Cartagena, COL; 3 Dentistry Program, Universidad del Sinú, Cartagena Sectional, Cartagena, COL; 4 Department of Basic Sciences, Faculty of Dentistry, University of Antioquia, Medellin, COL

**Keywords:** diagnosis, hamartoma, mouth diseases, neurilemmoma, ranula

## Abstract

Oral lesions with similar clinical presentation often conceal distinct histopathological profiles, creating diagnostic challenges and potential risks of misdiagnosis. This report presents the cases of three patients whose oral lesions appeared macroscopically alike but revealed markedly different microscopic characteristics. The first case involved a 20-year-old man with a persistent lower lip lesion that had recurred after two prior interventions; histopathological analysis confirmed a mucocele characterized by mucin extravasation and chronic inflammatory infiltrate. The second case was of a 68-year-old woman with a dorsal tongue mass that produced discomfort in speech and mastication; biopsy revealed a benign schwannoma with the classic biphasic Antoni A and B pattern, encapsulated and without malignant features. The third case was of a 65-year-old woman with a slow-growing, well-circumscribed lesion in the lower lip, which histology identified as a fibrovascular lipomatous hamartoma composed of mature adipose tissue, smooth muscle fibers, and abundant vascular elements. Despite their overlapping clinical appearance, these entities differ substantially in etiology, biological behavior, and treatment implications. The findings underscore the indispensable role of histopathological examination in establishing accurate diagnosis, guiding appropriate surgical management, and preventing recurrence. By demonstrating how macroscopic similarities may mask microscopic diversity, this series highlights the educational and clinical importance of systematic biopsy and histological evaluation in contemporary oral pathology practice.

## Introduction

Histopathological examination serves as the cornerstone of definitive diagnosis in oral pathology, enabling clinicians to move beyond presumptive diagnoses based solely on clinical appearance [1]. The integration of clinical findings with microscopic analysis is particularly crucial when evaluating oral diseases that present with similar macroscopic characteristics but exhibit distinct microscopic features.

Building upon evidence-based dentistry principles, the development of histopathological interpretation skills has become fundamental for achieving accurate diagnoses and establishing appropriate treatment protocols [2]. This approach directly impacts treatment planning, prognosis determination, and patient outcomes [3], especially when dealing with lesions sharing similar clinical presentations.

Among oral pathologies, mucocele, schwannoma, and hamartoma represent three distinct entities that can present with remarkably similar clinical features, creating significant diagnostic challenges [4]. Mucocele stands as the most frequently encountered non-neoplastic lesion of minor salivary glands, typically associated with traumatic etiology and maximum incidence in the second decade of life [5]. Schwannomas represent benign neural neoplasms derived from Schwann cells, comprising approximately 1% of head and neck neoplasms [6,7]. Hamartomas represent developmental anomalies characterized by disorganized proliferation of indigenous tissues rather than true neoplasms [8-10].

The objective of this case series is to demonstrate how three pathologies with identical clinical presentations require histopathological differentiation, emphasizing the critical importance of tissue-based diagnosis in contemporary oral pathology practice.

## Case presentation

Case 1: mucocele

Clinical History

A 20-year-old male patient with no relevant medical history presented at the maxillofacial surgery service with a nodular lesion on the lower lip with four years of evolution. The lesion had previously undergone two surgical extraction procedures with puncture, but recurrence occurred each time. The patient reported no associated pain, though he experienced occasional discomfort during eating and speaking. There was no history of recent trauma, though the patient acknowledged chronic lip biting habits. No family history of similar lesions was reported.

Clinical Findings

**Figure 1:**
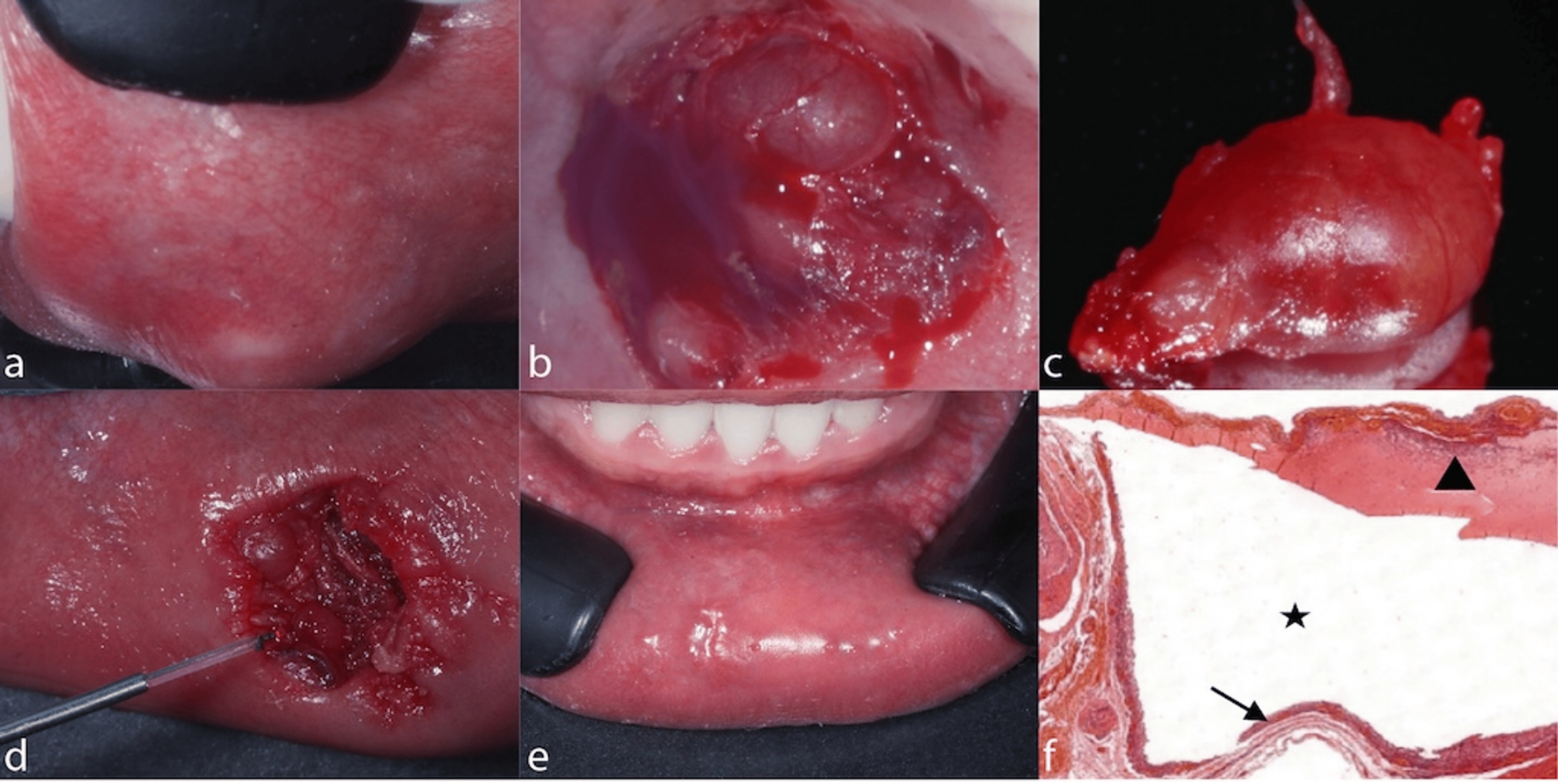
Mucocele surgical sequence and histopathology (Case 1) a. Initial clinical photograph of lower lip lesion showing a translucent, fluctuant nodule in the midline. b. Incision and surgical approach to the lesion with cold scalpel. c. Complete lesion excision for histopathological examination. d. Hemostasis and disinfection performed with diode laser. e. Suturing and application of tissue sealant. f. Microscopic view showing a cavity delimited in the connective tissue and submucosal layer (black star), causing notable elevation of the mucosa with epithelial thinning (black arrow). The presence of mucin accumulation and inflammatory infiltrate is evident (black triangle) (H&E staining, original magnification 100x).

The lesion was located in the midline of the lower lip, presenting exophytic characteristics and remaining asymptomatic throughout the evaluation period (Figure 1a). The lesion maintained the same color as the surrounding mucosa, appearing slightly translucent under clinical examination. The approximate dimensions measured 10 mm x 12 mm, and the lesion demonstrated a fluctuant consistency on palpation, suggesting fluid content. The surface appeared smooth and intact, without ulceration or secondary infection. Surrounding tissues showed no signs of inflammation or induration.

Key Clinical Features Suggesting Mucocele

The combination of lower lip location, translucent appearance, fluctuant consistency, history of trauma (chronic lip biting), and recurrence after incomplete excision strongly suggested a mucocele diagnosis. The bluish-translucent color and soft, fluctuant nature are pathognomonic features that distinguish mucoceles from other oral lesions.

Diagnostic Impression

Given the lesion's history, clinical appearance, and previous surgical interventions with recurrence, a comprehensive differential diagnosis was established, including: (i) Mucocele (most likely given the history and clinical presentation), (ii) Fibroma (considering the firm consistency in some areas), and (iii) Mucous retention cyst (given the fluctuant nature and location).

Treatment Plan

After obtaining written informed consent, a complete excisional biopsy was performed under local anesthesia using lidocaine 2% with epinephrine 1:100,000 (Figure 1b). The surgical approach involved careful dissection to include the underlying minor salivary glands to prevent recurrence (Figure 1c). Hemostasis was achieved using a diode laser (Figure 1d), and closure was performed with resorbable sutures (Figure 1e). The specimen was immediately fixed in 10% formalin and sent for histopathological examination.

Microscopic Description

Histological sections revealed minor salivary gland acini demonstrating marked edema and recent stromal hemorrhage (Figure 1f). The predominant finding was a prominently dilated duct containing mucous material in its lumen, characteristic of ductal obstruction. Multiple accumulations of neutrophils and histiocytes with foamy cytoplasm were observed throughout the specimen. The surrounding connective tissue showed a chronic inflammatory infiltrate with lymphocytes and plasma cells. No epithelial lining was observed around the mucin collection, confirming extravasation rather than retention.

Histopathological Diagnosis

The diagnosis was minor salivary gland mucocele with associated chronic inflammation.

Follow-up

The patient showed complete healing at one-month follow-up with no signs of recurrence at the postoperative, six-month follow-up.

Case 2 - schwannoma

Clinical History

A 68-year-old female patient presented with a lesion on the dorsal surface of the tongue with approximately eight months of evolution. The lesion was painless but caused progressive discomfort and difficulty with tongue movement, affecting speech articulation and mastication. The patient denied any history of trauma to the area and had no relevant personal or family medical history. She was edentulous in the maxilla and mandible, using complete dentures that were well-fitted and caused no apparent trauma to the lingual area.

Clinical Findings

**Figure 2:**
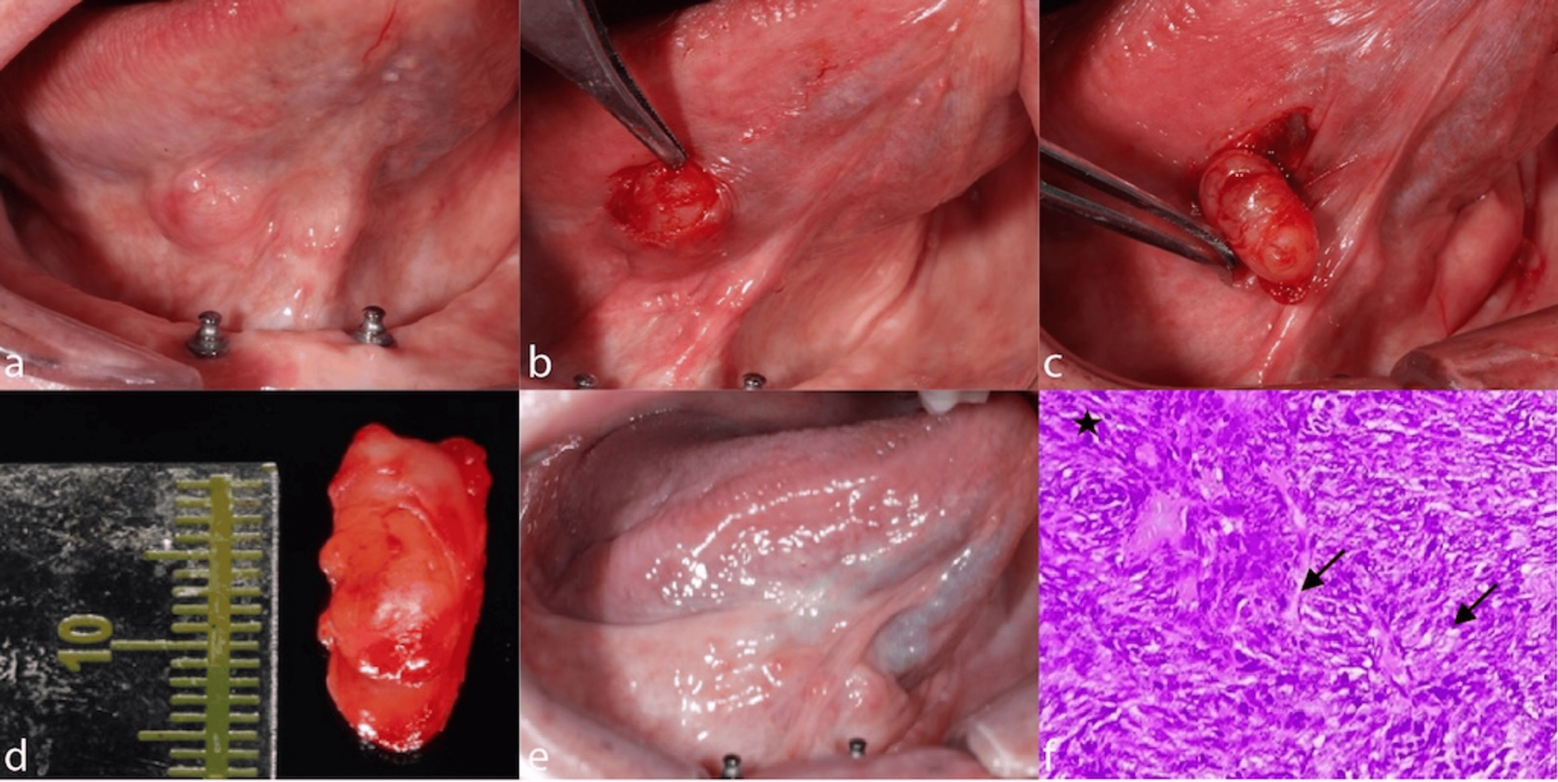
Schwannoma surgical sequence and histopathology (Case 2) a. Exophytic lesion on the right ventral surface of the tongue. b. Cold scalpel incision and careful lesion debridement. c. Complete lesion removal. d. Healing process of the excisional biopsy site showing satisfactory recovery. e. Lesion measurements for pathological examination. f. Nodular lesion in tissue with clear, encapsulated boundaries, composed of fusiform cells. Areas with high cellular density (Antoni A areas) (black star), and low cellular density (Antoni B areas) (black arrow) are distinguished, characteristic of schwannoma (H&E staining, original magnification 200x).

Clinical examination revealed normoglossia and normochelia with palatal mucosa showing no color changes or pathological alterations. The lesion appeared as an indurated, exophytic mass on the dorsal surface of the tongue (Figure 2a), measuring approximately 8 x 6 mm. The mass was mobile during palpation but non-pedunculated, firmly attached to the underlying lingual muscle. The surface appeared smooth with intact epithelium and normal coloration. No associated lymphadenopathy was detected during cervical palpation.

Key Clinical Features Suggesting Schwannoma

The firm, well-circumscribed nature of the lesion, its attachment to underlying muscle tissue, slow growth pattern over eight months, and absence of surface ulceration or color changes are characteristic features of schwannoma. The functional impairment (speech and mastication difficulties) without pain is typical of neural tumors.

Treatment Plan

Following informed consent, a complete excisional biopsy was recommended and performed due to the lesion size, location, and potential for malignancy, given the patient's age. The procedure was conducted under local anesthesia with careful attention to functional preservation of lingual movement (Figure 2b). The surgical approach involved minimal tissue sacrifice while ensuring complete lesion removal with adequate margins (Figure 2c).

Microscopic Description

Histological examination revealed a well-delimited and encapsulated nodular tissue lesion composed predominantly of fusiform cells arranged in fascicles (Figure 2f). The lesion demonstrated characteristic areas of high cellularity (Antoni A areas) alternating with regions of low cellularity (Antoni B areas), pathognomonic features of schwannoma. No cellular atypia, mitotic activity, or necrosis was identified throughout the specimen. The capsule appeared intact, and no infiltration into surrounding tissues was observed.

Histopathological Diagnosis

The diagnosis was benign schwannoma of the tongue.

Follow-up

Complete functional recovery was achieved with no residual speech or movement difficulties. The healing process was satisfactory (Figure 2e), and no recurrence was observed at the 12-month follow-up.

Case 3 - hamartoma

Clinical History

A 65-year-old female patient presented with a slow-growing lesion in the lower lip region with approximately two years of evolution. The lesion was completely asymptomatic and was discovered incidentally during a routine dental examination. The patient had no history of trauma to the area and no relevant medical or family history. She was concerned primarily about the cosmetic appearance and potential for malignant transformation.

Clinical Findings

**Figure 3:**
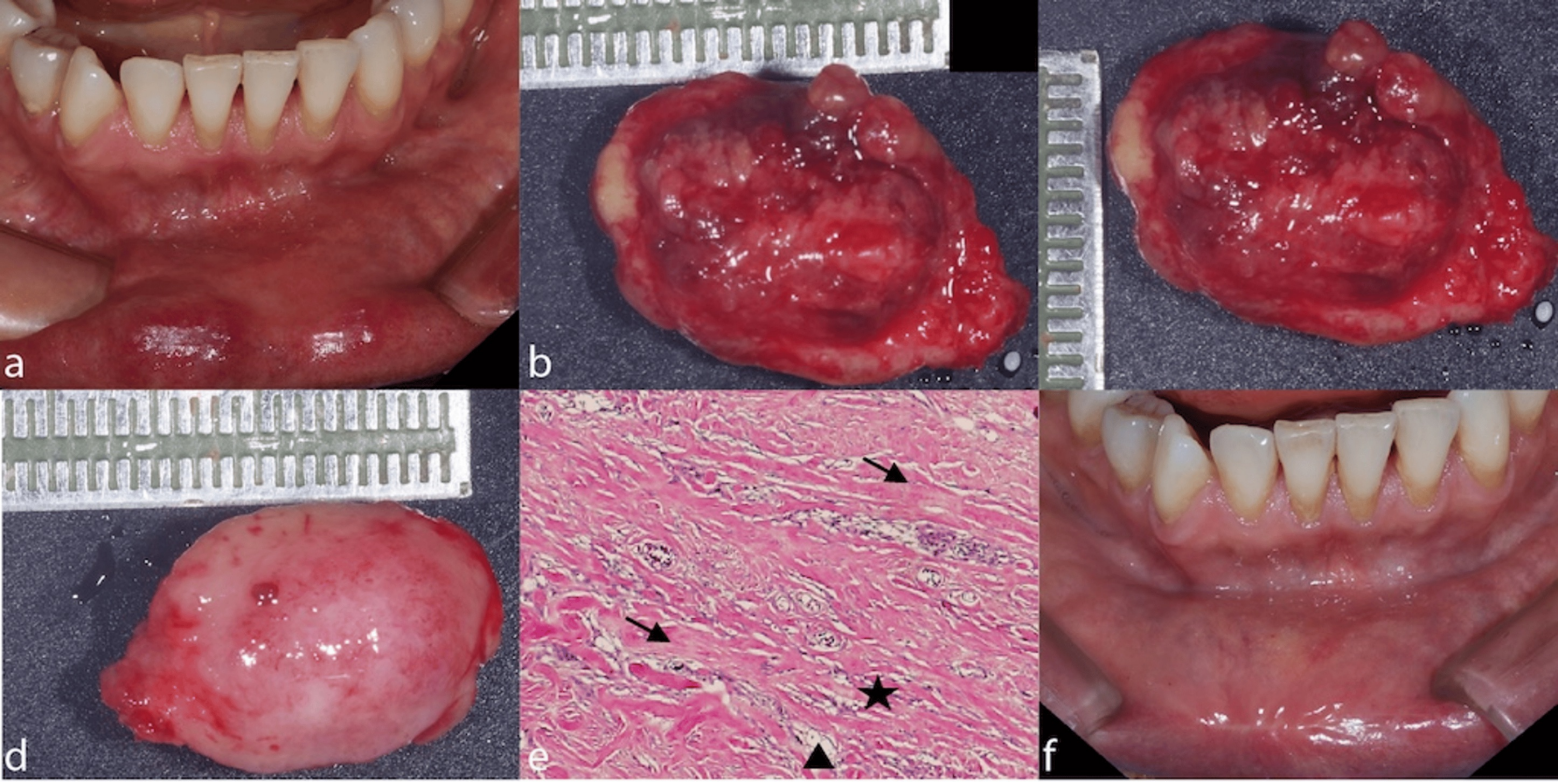
Hamartoma surgical sequence and histopathology (Case 3) a. Exophytic lesion on the right lower lip. b. Internal pathology specimen measuring 15 mm in length. c. Internal pathology specimen measuring 12 mm in width. d. External pathology specimen with lip skin, length measurement. e. Healed lip showing excellent cosmetic result. f. Fibroblastic proliferation with multiple capillary blood vessels and fibrolipomatous tissue (black star) with mature adipose cells (black triangle). These elements are intertwined with benign smooth muscle fibers in the analyzed sample (black arrow), characteristic of fibrovascular lipomatous hamartoma (H&E staining, original magnification 100x).

Physical examination revealed an exophytic lesion measuring approximately 15 x 15 mm, firmly adherent to deep tissue planes (Figure 3a). The lesion presented with slight induration and mild erythema of the overlying lip skin. Palpation elicited minimal tenderness without producing ischemia or blanching. The lesion appeared well-circumscribed with no evidence of rapid growth or surface ulceration.

Key Clinical Features Suggesting Hamartoma

The slow growth over two years, well-circumscribed borders, firm consistency, and mixed tissue appearance (involving both mucosal and cutaneous components) are characteristic of hamartoma. The absence of symptoms and the heterogeneous composition on palpation distinguish it from other benign lesions.

Treatment Plan

After obtaining proper informed consent, a complete excisional biopsy was performed using the cold scalpel technique, including both mucosal and cutaneous components to ensure complete removal. The procedure involved careful layer-by-layer dissection to preserve cosmetic appearance while achieving clear margins. Hemostasis was accomplished using a diode laser, and closure was performed in multiple layers using appropriate suture materials. The specimen was carefully measured from both internal (Figure 3b, 3c) and external aspects (Figure 3d) for comprehensive pathological evaluation.

Microscopic Description

Examination revealed mucosa covered by stratified squamous epithelium with reactive changes resembling seborrheic keratosis-like features and focal epithelial atrophy (Figure 3e). The underlying tissue demonstrated extensive fibroblastic proliferation with numerous capillary and small-caliber blood vessels filled with erythrocytes. A characteristic fibrolipomatous proliferation was identified, containing well-differentiated mature adipose cells intermixed with vascular structures and benign smooth muscle fibers. Minor salivary gland lobules showed focal epithelial atrophy with minimal chronic inflammatory infiltrate.

Histopathological Diagnosis

The diagnosis was of fibrovascular lipomatous hamartoma.

Follow-up

Excellent cosmetic healing was achieved with patient satisfaction (Figure 3f) and no evidence of recurrence at the one-year follow-up.

## Discussion

The integration of clinical evaluation with definitive histopathological analysis represents the cornerstone of accurate diagnosis in oral pathology [1]. This case series demonstrates that morphological similarity does not equate to diagnostic equivalence, particularly when dealing with lesions presenting identical clinical characteristics but exhibiting completely different microscopic features. Each pathological entity demonstrates distinct characteristics that guide differential diagnosis.

Mucoceles represent the most common non-neoplastic salivary gland lesions, with pathogenesis involving mechanical trauma causing ductal disruption [11,12]. The extravasation type, as demonstrated in our first case, is characterized by the absence of epithelial lining around mucin collection, distinguishing it from retention cysts [6]. Complete excision, including associated minor salivary glands, is essential to prevent recurrence [8].

Schwannomas present unique diagnostic challenges as they comprise less than 1% of oral soft tissue neoplasms [13]. The characteristic biphasic pattern of Antoni A and Antoni B areas represents the pathognomonic feature distinguishing schwannomas from other spindle cell lesions [7]. Their encapsulated nature and lack of malignant potential make complete excision curative, though careful surgical technique is essential when located near major nerve branches. These benign neural tumors can occasionally mimic other common oral lesions, including pyogenic granulomas and fibromas [14].

Hamartomas represent developmental anomalies characterized by disorganized proliferation of indigenous tissues [9]. The fibrovascular lipomatous variant combines multiple tissue types in a benign configuration, with the presence of mature adipose tissue, smooth muscle fibers, and vascular elements confirming the hamartomatous nature [10].

The clinical similarities of these pathologies demonstrate that establishing a definitive diagnosis based solely on macroscopic characteristics risks misdiagnosis or inappropriate treatment planning [15]. Without histopathological confirmation, clinicians might default to diagnosing the most commonly encountered lesion, potentially leading to inadequate treatment or unexpected recurrence.

## Conclusions

Clinical similarity does not guarantee diagnostic equivalence. These three cases demonstrate that pathologies affecting the oral cavity require differentiation beyond simple morphological assessment, as identical macroscopic presentations may conceal completely different microscopic characteristics and etiologies. Histopathological examination must be considered mandatory following all surgical interventions for oral lesions, regardless of presumed benign nature. This approach guarantees definitive diagnosis, enables appropriate treatment planning, and ensures accurate prognosis determination.

The successful outcomes achieved in all cases reflect the value of evidence-based diagnostic approaches that prioritize comprehensive differential diagnosis formulation, complete surgical excision with appropriate margins, and systematic histopathological evaluation in contemporary oral pathology practice.
